# Supplementary material for: Chronic bee paralysis as a serious emerging threat to honey bees
Source: Nat Commun. 2020 May 1;11:2164. doi: 10.1038/s41467-020-15919-0 (PMC7195492; doi:10.1038/s41467-020-15919-0)
Supplement: Supplementary file 1 — Supplementary Information [file 41467_2020_15919_MOESM1_ESM.pdf]

Chronic bee paralysis as a serious emerging threat to honey bees

Budge *et al.*

Supplementary Information

Disease data supplementary observations

The mean number of apiary visits in each year was 7,795 (SD=1,355.9), although these rose to a peak of 8,926 in 2009 before progressively declining to 6,028 in 2017 (Supplementary Figure S1A). The mean number of colonies in an apiary visited in England and Wales was 5.89 (SD 10.76), skewed to the left with a long tail of apiarists with 20 or more colonies. In addition, 24% (5,797) of visited beekeepers had more than one apiary visited.

The proportion of apiary visits to professional beekeepers that had imported honey bees increased significantly during the study period with 9% recording imports in 2006 and 30% in 2017. The increase through time was significant at 2.5% per year (t= 8.017, P=1.16e-05). Whilst visits to amateur beekeepers indicated they also imported honey bees, the increase in importations over the period, although significant (t=4.228, P= 0.00175), was only 0.07% per year.

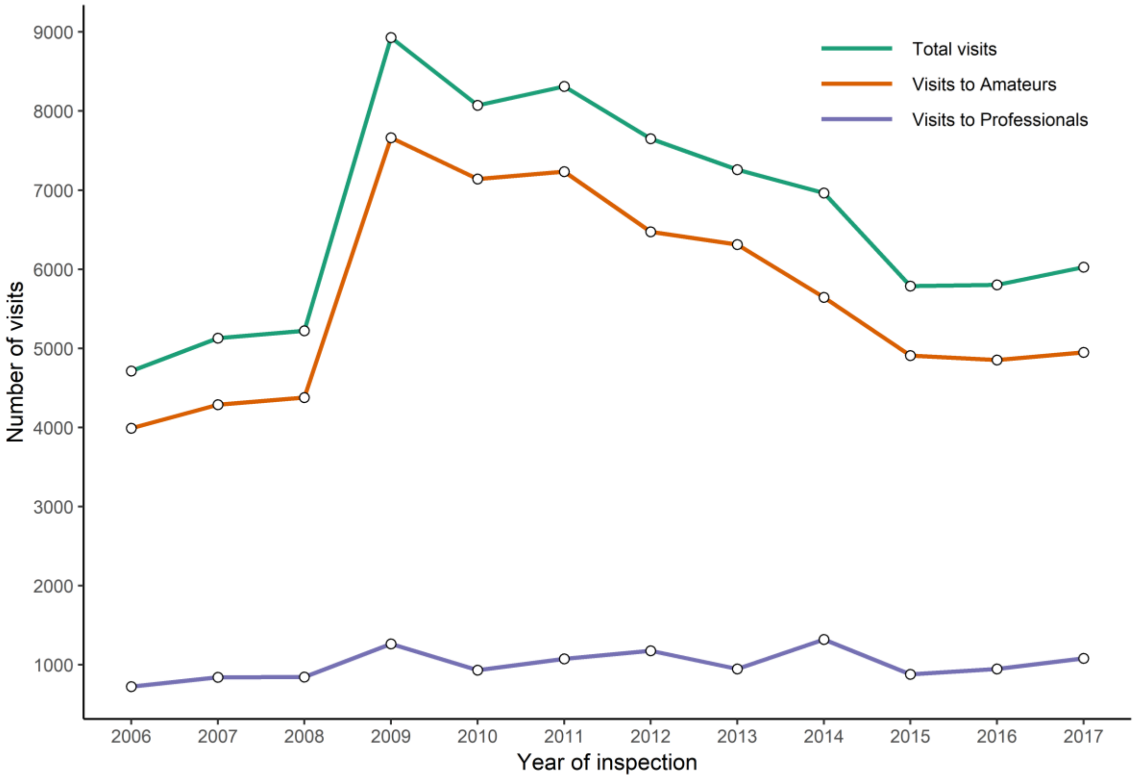

Supplementary Figure 1 Number of apiary visits made by honey bee health inspectors between 2006 and 2017. Amateur beekeepers are defined as those that own fewer than 40 colonies and professional beekeepers those that own 40 or more colonies.

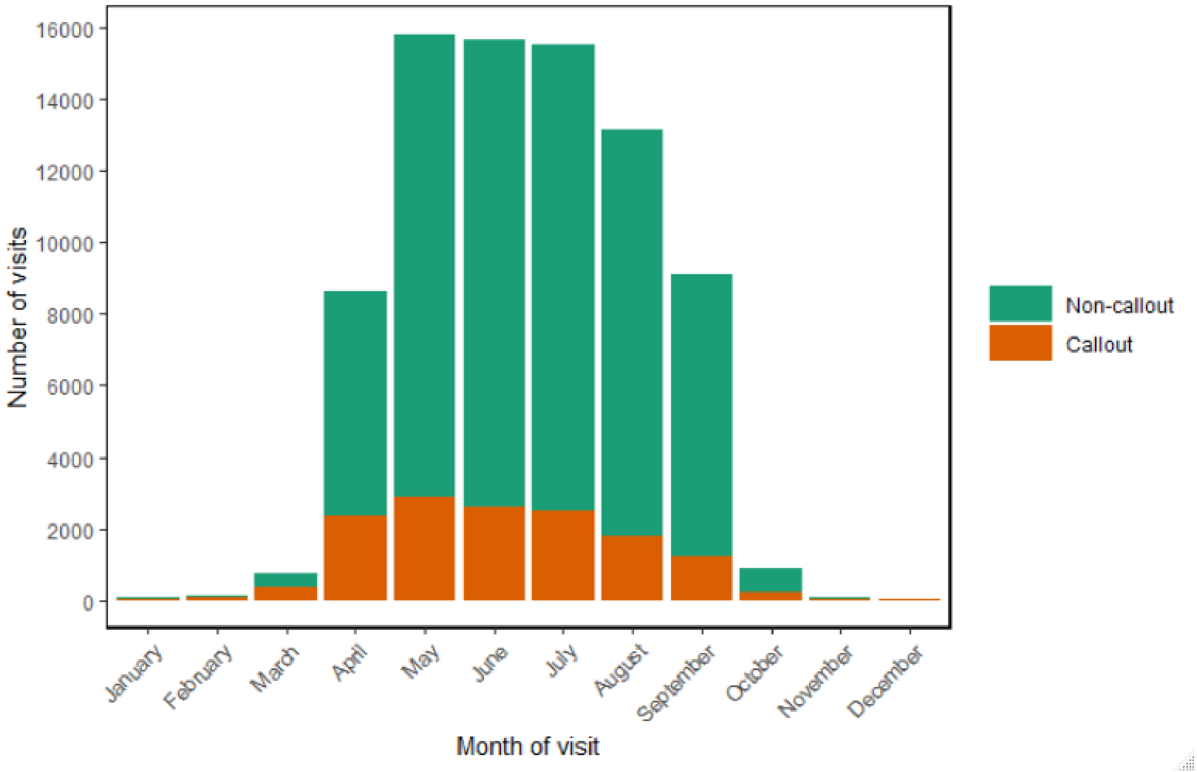

Supplementary Figure 2 Number of callout and non-callout visits in each month indicating the seasonal nature of honey bee colony health visits.

Supplementary Table 1 Annual number of honey bee importers and honey bee imports.

	2007	2008	2009	2010	2011	2012	2013	2014	2015	2016	2017	Total
# Importers	43	62	52	58	55	60	86	82	104	112	115	327*
# Imports	10,549	9,875	10,840	10,173	6,892	8,889	12,807	12,991	13,842	16,303	17,585	130,746

* Represents the number of unique importers during the period.
