# Supplementary material for: Chronic bee paralysis as a serious emerging threat to honey bees
Source: Nat Commun. 2020 May 1;11:2164. doi: 10.1038/s41467-020-15919-0 (PMC7195492; doi:10.1038/s41467-020-15919-0)
Supplement: Supplementary file 3 — Reporting Summary [file 41467_2020_15919_MOESM3_ESM.pdf]

nature research

Corresponding author(s): Giles Budge
Last updated by author(s): Mar 24, 2020

Reporting Summary

Nature Research wishes to improve the reproducibility of the work that we publish. This form provides structure for consistency and transparency in reporting. For further information on Nature Research policies, see Authors & Referees and the Editorial Policy Checklist .

Statistics

For all statistical analyses, confirm that the following items are present in the figure legend, table legend, main text, or Methods section.

n/a	Confirmed	
☐	☒	The exact sample size (n) for each experimental group/condition, given as a discrete number and unit of measurement
☐	☒	A statement on whether measurements were taken from distinct samples or whether the same sample was measured repeatedly
☐	☒	The statistical test(s) used AND whether they are one- or two-sided<br>*Only common tests should be described solely by name; describe more complex techniques in the Methods section.*
☐	☒	A description of all covariates tested
☐	☒	A description of any assumptions or corrections, such as tests of normality and adjustment for multiple comparisons
☐	☒	A full description of the statistical parameters including central tendency (e.g. means) or other basic estimates (e.g. regression coefficient) AND variation (e.g. standard deviation) or associated estimates of uncertainty (e.g. confidence intervals)
☐	☒	For null hypothesis testing, the test statistic (e.g. F, t, r) with confidence intervals, effect sizes, degrees of freedom and P value noted<br>*Give P values as exact values whenever suitable.*
☒	☐	For Bayesian analysis, information on the choice of priors and Markov chain Monte Carlo settings
☒	☐	For hierarchical and complex designs, identification of the appropriate level for tests and full reporting of outcomes
☒	☐	Estimates of effect sizes (e.g. Cohen's d, Pearson's r), indicating how they were calculated

Our web collection on statistics for biologists contains articles on many of the points above.

Software and code

Policy information about availability of computer code

Data collection	Case data were not collected using software, instead they were downloaded from government SQL database via a licence agreement.
Data analysis	All analyses were performed using R version 3.6.0. Packages are stated in the manuscript text.

For manuscripts utilizing custom algorithms or software that are central to the research but not yet described in published literature, software must be made available to editors/reviewers. We strongly encourage code deposition in a community repository (e.g. GitHub). See the Nature Research guidelines for submitting code & software for further information.

Data

Policy information about availability of data

All manuscripts must include a data availability statement. This statement should provide the following information, where applicable:

- Accession codes, unique identifiers, or web links for publicly available datasets
- A list of figures that have associated raw data
- A description of any restrictions on data availability

Visit data contain personal information and were obtained under a restricted data confidentiality license agreement from the Animal and Plant Health Agency (contact enquiries@apha.gsi.gov.uk). Honey bee import data were obtained under license from the EU TRACES database and are presented in Fig. 5 and Supplementary Table 1. Real-time RT qPCR data is provided as a Supplementary Data file. The source data underlying Figs 1a-b, 2, 3, 6 and Supplementary Figs 1 and 2 are all provided as a Source Data file.

Field-specific reporting

Please select the one below that is the best fit for your research. If you are not sure, read the appropriate sections before making your selection.

Ecological, evolutionary & environmental sciences study design

All studies must disclose on these points even when the disclosure is negative.

Study description	We determined the location of chronic bee paralysis using information from an existing government honey bee health inspection regime. We explored metadata from each apiary visit to assign, location, county, beekeeper type (amateur or professional), and import history. We then obtained honey bee import data from the EU Trade Control and Expert System (TRACES) that had been deposited into a government database. In 2017, we requested that NBU inspectors and bee farmers collect adult honey bee samples from apiaries that were either asymptomatic (n=24) or symptomatic (n=25) for chronic bee paralysis virus. Adult bees showing symptoms of paralysis were sampled from symptomatic colonies and healthy returning foragers were sampled from asymptomatic colonies. We compared virus levels in these adult bees.
Research sample	We did not subsample the data, instead we sampled the entire population of data available.
Sampling strategy	We did not subsample the data, instead we sampled the entire population of data available.
Data collection	Chronic bee case data were recorded by the National Bee Unit based at the Animal and Plant Health Agency. Data were gathered on behalf of the Department for Environment Food and Rural Affairs and Welsh government to fulfil honey bee health surveillance programmes. Apiary visit data were recorded by appointed bee inspectors of the National Bee Unit when they visited apiaries and assessed the health of the honey bee colonies therein. Import data were obtained under license through the EU TRACES system. RT qPCR data were gathered by Dr. Nicola Simcock as described in the methods.
Timing and spatial scale	Apiary visit data with the appropriate metadata for our study were collected from 2006 through to 2017 with between 4,714 and 8,926 observations per annum. Except for seasonality if inspections, there are no gaps in the visit data and their spatial scale is across England and Wales. Honey bee import data were gathered between 2006 and 2017, with no gaps and honey bee imports were received from 25 countries (as stated in the manuscript).
Data exclusions	Some apiary visit data fell outside the land mass of the UK and so was excluded due to location recording errors (40 of 79,873). Similarly, 2% of the honey bee imports could not be assigned spatially and so were also excluded.
Reproducibility	This is not applicable because we used the entire available datasets.
Randomization	This is not applicable because we used the entire available datasets.
Blinding	We had no previous assumptions about where chronic bee paralysis might occur when assessing the visit data for the presence of the disease, therefore this is not applicable.

Did the study involve field work? ☒ Yes ☐ No

Field work, collection and transport

Field conditions	We collected samples from 24 symptomatic and 23 asymptomatic apiaries as described in the methods. Field visits would have been during periods of fine weather (temperature>12C; no rain) to avoid unnecessary disturbance to the honey bee colonies.
Location	Apiaries locations were from 25 English counties (Buckinghamshire, Cambridgeshire, Cheshire, Cumbria, Devon, Dorset, Essex, Gloucestershire, Greater Manchester, Hampshire, Hertfordshire, Kent, Lancashire, Lincolnshire, North Yorkshire, Oxfordshire, Staffordshire, Suffolk, Surrey, Tyne & Wear, Warwickshire, West Midlands, West Sussex, West Yorkshire, Worcestershire) and 5 Welsh counties (Ceredigion, Clwyd, Dyfed, Gwent, Powys).
Access and import/export	Apiaries were accessed with the permission of the beekeeper. No licenses were required for sampling because chronic bee paralysis is not a statutory notifiable disease. No materials crossed any border and so no import or export licenses were required.
Disturbance	Samples were collected during routine honey bee colony health assessments and so no additional colony disturbances were required. All visits would have been completed during periods of fine weather (see above) to avoid unnecessary disturbance to the honey bee colonies.

Reporting for specific materials, systems and methods

We require information from authors about some types of materials, experimental systems and methods used in many studies. Here, indicate whether each material, system or method listed is relevant to your study. If you are not sure if a list item applies to your research, read the appropriate section before selecting a response.

Materials & experimental systems

n/a	Involved in the study
☒	☐ Antibodies
☒	☐ Eukaryotic cell lines
☒	☐ Palaeontology
☒	☐ Animals and other organisms
☒	☐ Human research participants
☒	☐ Clinical data

Methods

n/a	Involved in the study
☒	☐ ChIP-seq
☒	☐ Flow cytometry
☒	☐ MRI-based neuroimaging
